# Severe Periodontitis Increases the Risk of Oral Frailty: A Six-Year Follow-Up Study from Kashiwa Cohort Study

**DOI:** 10.3390/geriatrics8010025

**Published:** 2023-02-13

**Authors:** Misa Nishimoto, Tomoki Tanaka, Hirohiko Hirano, Yutaka Watanabe, Yuki Ohara, Maki Shirobe, Katsuya Iijima

**Affiliations:** 1 Institute of Gerontology, The University of Tokyo, Tokyo 113-8656, Japan; 2 R&D Department, Sunstar Inc., Tokyo 105-0014, Japan; 3 Tokyo Metropolitan Institute for Geriatrics and Gerontology, Tokyo 173-0015, Japan; 4 Gerodontology, Department of Oral Health Science, Faculty of Dental Medicine, Hokkaido University, Sapporo 060-8586, Japan; 5 Institute for Future Initiatives, The University of Tokyo, Tokyo 113-0033, Japan

**Keywords:** older adults, oral health, oral frailty, periodontal disease

## Abstract

Oral frailty, overlapping a decline in multi-faceted oral functions and often seen in older adults, increases risks of adverse health outcomes, thereby necessitating earlier measures. Tooth loss, a major element of oral frailty, is mainly caused by periodontal disease and is an irreversible event. Therefore, this study aimed to clarify whether advanced periodontal disease increases the risks of “new-onset” oral frailty through a longitudinal analysis based on the 2012 baseline survey of the Kashiwa cohort and the follow-up assessments conducted in 2013, 2014, 2016, and 2018. The participants were disability-free, non-orally frail older adults living in Kashiwa City. Of the 1234 participants (72.2 ± 5.1 years old; 50.8% men) analyzed in this study, oral frailty occurred in 23.1% within the six-year period. The group with Community Periodontal Index (CPI) ≥ 3 at baseline had no significant difference in the risk of oral frailty compared with CPI ≤ 2; however, CPI4 at baseline was related to the increased risk of oral frailty compared with CPI ≤ 3 (an adjusted hazard ratio (95% confidence interval): 1.42 (1.12–1.81). Conclusively, severe periodontitis (CPI4) might be associated with new-onset oral frailty, suggesting that prevention of periodontal disease could contribute to oral frailty prevention.

## 1. Introduction

Population aging is a global challenge, and initiatives seeking to extend health throughout our lives (as life expectancy increases) are in demand. Frailty is a state of decreased physiological reserve and increased vulnerability to stressors and is a common health condition that leads to adverse health outcomes in older adults and threatens healthy aging [1,2].

The relationship between poor oral health and frailty has been reported in previous studies [3,4]. The oral cavity consists not only of teeth but also other parts that assist in chewing, swallowing, and communicating. Therefore, it is important to maintain the number of teeth as well as various oral functions in older adults. Prevention methods for tooth loss have already been established, but there are still few reports on how to prevent the decline of oral functions. Oral frailty is defined as the combination of aging-associated tooth loss and the decline of multi-faceted oral functions [3,4]. We reported that community-dwelling older adults with oral frailty face an increased risk of physical frailty, sarcopenia, need for long-term care, and mortality [4]. It has also been reported that older adults with oral frailty have lower gait performance [5], poorer nutritional status [6,7], higher rates of eating alone [8], and lower food satisfaction [9]. Thus, oral frailty is thought to have a negative impact not only on physical health but also on psychological, social, and even quality of life (QOL).

While understanding how to prevent oral frailty and alleviate its symptoms is a challenge, it can be conceivably achieved by applying a two-sided approach: (1) maintaining healthy teeth and (2) heightening strength in the muscles involved in oral functions related to ingestion, swallowing, and speaking. It is possible to restore oral health that has deteriorated to some extent, but tooth loss presents an especially difficult challenge as a factor of oral frailty because it is irreversible. The leading cause of tooth loss in old age is periodontal disease, a chronic condition caused by bacterial infection and resulting in an adverse effect on oral functions due to the deterioration of periodontal tissue. However, it has not been ascertained whether periodontal disease is a risk factor for oral frailty.

Oral functional training (involving use of the tongue, mastication, etc.) for community-dwelling older adults represents one type of intervention that has reported some success in preventing oral frailty and alleviating its symptoms, but it is generally difficult for people to incorporate this as a usable approach in their daily lives [10,11]. On the other hand, daily oral hygiene is fundamental to the prevention of periodontal disease, and there are a wide variety of self-care products available to consumers for this purpose. Therefore, by clarifying that the prevention of periodontal disease also leads to the prevention of oral frailty, it is possible to further emphasize the significance of conventional oral hygiene as one of the ways to prevent oral frailty. 

In this context, this study aims to examine whether periodontal disease is associated not only with a decrease in the number of remaining teeth but also with the increased risk of new-onset oral frailty among community-dwelling older adults.

## 2. Materials and Methods

### 2.1. Setting and Participants

We used data from the Kashiwa study, which identified factors affecting the prolongation of healthy life expectancy in a longitudinal cohort of community-dwelling older adults (aged 65 years and older) [4]. The participants of this study were enrolled in 2012, and the baseline survey was conducted in the same year. Invitations to participate in the study were mailed to 12,000 residents who were randomly selected from the resident registry (comprising intermingled urban and rural communities) in Kashiwa City, Chiba Prefecture, Japan. A total of 2044 older adults (1013 men and 1031 women) agreed to participate in the study. The age and sex distributions of the study sample were representative of all older adults registered as residing in Kashiwa City.

Baseline data were collected at welfare centers and community centers between September and November 2012. In total, four follow-up assessments using the same methods as the baseline survey were conducted in 2013, 2014, 2016, and 2018. Inclusion criteria for participation in our study using follow-up surveys were as follows: Participants must (1) be community-dwelling and 65 years of age or older and (2) have no certification from the long-term care system as disabled. Exclusion criteria comprised older adults (1) with oral frailty at the baseline survey; (2) with complete edentulism at the baseline survey; and (3) who did not participate in any of the follow-up assessments. 

### 2.2. Measures

The oral examination items were the components of oral frailty, periodontal health status, number of functional teeth, and oral wettability. All examinations were conducted by trained dentists and dental hygienists with clinical practice experience. Workshops were held to train all dental staff in the assessment of the outcome measures.

### 2.3. Oral Frailty

A diagnosis of oral frailty was based on six criteria: (1) number of natural teeth; (2) chewing ability as an indicator of general masticatory performance; (3) maximum tongue pressure; (4) articulatory oral motor skill (“ta” sound); (5) subjective difficulties in eating hard foods; and (6) subjective difficulties in swallowing. We defined “oral non-frailty” as having no poor status for any of the above six criteria, while “oral frailty” was defined as having a poor status for three or more criteria [4]. 

“New-onset” oral frailty was defined as the first documentation of oral frailty since completion of the baseline survey. The follow-up period was defined as the number of years between the time of new-onset and completion of the baseline survey. On the other hand, “no-onset” oral frailty was defined as not meeting the criteria for oral frailty during the six-year follow-up period.

The number of natural teeth was assessed by dental professionals using the Zsig-mondy–Palmer system [12]. As almost all Japanese dental hygienists use this system, the inter-examiner calibration was deemed to be sufficient. Participants with less than 19 natural teeth were categorized as having a poor dental status [4].

Chewing ability was measured using a color-changing chewing gum (Xylitol; Lotte, Tokyo, Japan), which changes from green to red with chewing. Participants were asked to chew the gum for 60 s in the same way that they would chew food. We evaluated the gum’s redness using a colorimeter (Color Reader CR-13; Konica Minolta, Tokyo, Japan) [13]. Participants with the a-value (redness or greenness) of less than 14.2 (for men) or 10.8 (for women) were categorized as having a poor general masticatory performance [4].

Tongue pressure was measured with a hand-held balloon probe and manometer (JMS tongue pressure measurement instrument; GC, Tokyo, Japan) [14]. Participants were asked to place the balloon on the anterior part of their palate and to raise their tongue to compress the balloon onto the palate as forcefully as possible. Participants exerting a force of less than 27.4 kPa (for men) or 26.5 kPa (for women) were categorized as having a poor tongue pressure [4].

Oral diadochokinesis (“ta”) was assessed as a measure of articulatory oral motor skill [15]. Participants were asked to articulate this syllable repetitively as quickly as possible for five seconds. This syllable allowed for assessment of the three major articulatory organs: the lips, the tongue tip, and the tongue dorsum. Articulation counts were measured using a digital counter (T.K.K.3350 digital counter; Takei Scientific Instruments Co., Ltd., Niigata, Japan), and oral diadochokinesis was calculated separately, also using this syllable, as the articulation count per second. Participants with articulation rates of less than 5.2 times/second (for men) or 5.4 times/second (for women) were categorized as having poor articulatory oral motor skill [4].

Subjective assessments were conducted to evaluate difficulties in eating and swallowing, using the Kihon Checklist (KCL) questionnaire, an instrument designed to assess risk of dependence for older adults [16]. The checklist includes questions such as, “Do you have any difficulties eating hard foods compared to 6 months ago (Yes or No)?”; “Have you choked on your tea or soup recently (Yes or No)?”; and “Do you often experience having a dry mouth (Yes or No)?”

### 2.4. Periodontal Health Status

Periodontal health status was evaluated based on the Community Periodontal Index (CPI) [17]. A total of 10 predetermined teeth (16, 17, 11, 26, 27, 31, 36, 37, 46, and 47) were each assigned the following scores: 0 (healthy periodontal condition); 1 (gingival bleeding); 2 (calculus and bleeding); 3 (shallow periodontal pockets 4–5 mm in depth); and 4 (deep periodontal pockets with a depth of 6 mm or more). The highest score was recorded as the individual’s CPI score. 

The CPI scores were then used to score participants’ periodontal assessments using the World Health Organization Community Periodontal Index probe: participants were categorized as having severe periodontitis if the CPI score was 4, and moderate periodontitis if the CPI score was 3. CPI scores of 0, 1, or 2 indicated the absence of periodontitis. Periodontal health status was dichotomized as either with/without moderate periodontitis (CPI Code ≤ 2/≥3) or with/without severe periodontitis (CPI Code ≤ 3/4).

### 2.5. Covariates

Demographic characteristics including age, sex, number of teeth, education, yearly income (obtained from public databases), body mass index (BMI), mini-mental state examination (MMSE) score, geriatric depression scale—15 (GDS-15) score, instrumental activities of daily living (IADL), current chronic conditions, oral wettability, and polypharmacy (≥6 medications) were collected. Oral wettability was measured tongue dorsum moisture using the Kiso-Wet tester (Kiso science, Yokohama, Japan), which is based on ultrathin-layer chromatography [18]. The measurements were made by placing the tester vertically on the tongue dorsum approximately 1 cm from the tip of the tongue for 10 s and recording the height of the moistened area. A height of <3 mm was classified as dry mouth.

### 2.6. Statistical Analysis

Differences in characteristics between participants with and without new-onset oral frailty were analyzed using the chi-square test for categorical data and the Student’s *t*-test for continuous data. The association between periodontal health status at baseline and new-onset oral frailty was analyzed using the chi-square test. In addition, the data were analyzed using the Cox proportional hazards model, and hazard ratios and 95% confidence intervals adjusted for covariates were calculated. All statistical analyses were performed using IBM SPSS statistics version 24.0 (IBM Japan, Tokyo, Japan). We considered a *p*-value of less than 0.05 as statistically significant.

## 3. Results

### 3.1. Study Participant Characteristics

**Figure 1:**
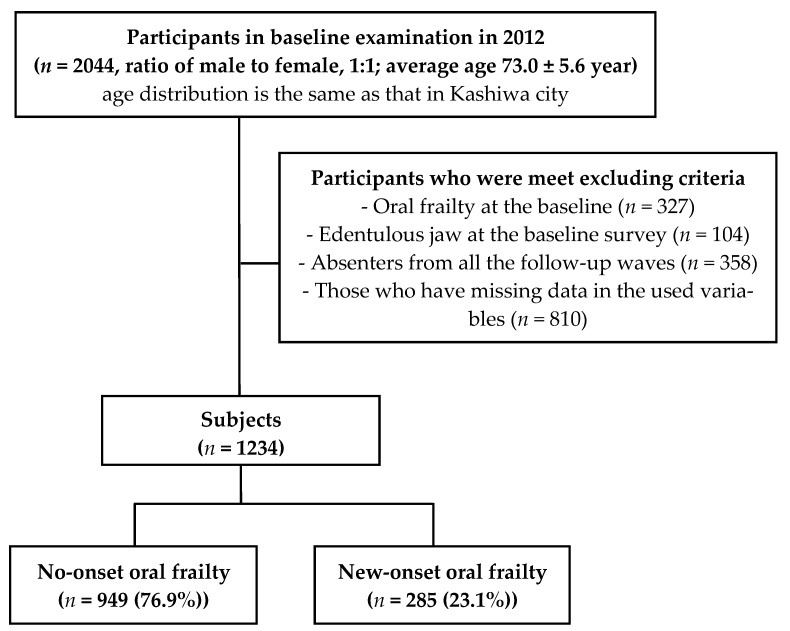
Study flowchart.

A total of 2044 older adults (1015 men and 1029 women) initially agreed to participate in the examinations and surveys. For the longitudinal analysis regarding new-onset oral frailty, we excluded those who presented with baseline oral frailty (*n* = 327) or edentulous jaw at the baseline survey (n = 104); those absent from all four of the follow-up waves (*n* = 358) and those who had missing data for the relevant variables (*n* = 810). Hence, a total of 1234 individuals (50.8% men; mean age at baseline 72.2 years) participated and contributed data for the analysis. Over the follow-up period (median year (interquartile range), 6 (4–6) years), 285 individuals (23.1%) had new-onset oral frailty (Figure 1).

### 3.2. Comparison of Basic Characteristics at Baseline with and without New-Onset Oral Frailty

The comparison between demographic variables and oral frailty status is shown in Table 1. The participants with new-onset oral frailty were significantly older (71.7 years vs. 73.8 years) (*p* < 0.001). Older adults with new-onset oral frailty were also significantly highly likely to have low cognitive function and depression (*p* = 0.021 and *p* = 0.009, respectively), and as a group, they had statistically significant higher rates than others of the chronic diseases diabetes mellitus, osteoporosis, and apoplexy (*p* = 0.035, *p* = 0.029, and *p* = 0.010, respectively). However, no significant differences were found between cases of new-onset oral frailty and sex, BMI, education, IADL, yearly income, or having the chronic conditions hypertension, dyslipidemia, malignant neoplasm, or heart disease.

### 3.3. Comparison of Periodontal Health Status at Baseline with and without New-Onset Oral Frailty

The comparison between periodontal health status at baseline and new-onset oral frailty status is shown in Table 2. At baseline, the breakdown of CPI scores for all participants as “CPI score, n (% of N)” was CPI0, 17 (1.4%); CPI1, 17 (1.4%); CPI2, 282 (22.8%); CPI3, 439 (35.5%); and CPI4, 480 (38.9%). There was no statistically significant difference between CPI scores and new-onset oral frailty.

### 3.4. Relationship between Periodontal Health Status and New-Onset Oral Frailty

The hazard ratios of new-onset oral frailty based on the periodontal health statuses are shown in Table 3. The Cox proportional hazards models revealed that participants with CPI ≥ 3 had no significant difference in the risk of oral frailty compared with those with CPI ≤ 2, whereas participants who had CPI4 had the increased risk of oral frailty compared with those with CPI ≤ 3 (an adjusted hazard ratio (95% confidence interval): 1.42 (1.12–1.81)).

## 4. Discussion

The present study assessed the decrease in the number of teeth as well as the multi-faceted decline in oral function (oral frailty) in community-dwelling older adults over a six-year period and found cases of new onset. We investigated the association between periodontal status at the baseline survey and new-onset oral frailty. The results showed that severe periodontitis increased the risk of new-onset oral frailty even after eliminating the effects of confounding factors.

The decrease in the number of remaining teeth and associated decline in chewing ability are some intermediary factors that may explain the relationship between periodontal disease and new-onset oral frailty [19,20]. Previous studies have reported that tooth mobility, tooth loss, and decreased occlusal support associated with periodontal disease affect masticatory function [19] and that advanced periodontal disease reduces masticatory ability even without tooth loss [20]. Thus, they reasonably support the results of our study: worsening of severe periodontitis causes not only tooth mobility or loss but also decreases masticatory function, thereby increasing the likelihood of onset to oral frailty. Pain on chewing due to severe periodontitis may have also contributed to decreased masticatory function and subjective chewing difficulty. Furthermore, in addition to the direct effects of severe periodontitis on oral function, periodontitis-related halitosis and degradation of aesthetics also affect social relationships [21,22]. Moreover, periodontal disease is also associated with depressive tendency and decline of cognitive function [23,24]. Since the relationship between these factors and oral frailty has also been revealed [4], indirect effects via psychological and/or social factors should also be considered. Accordingly, we hypothesize that severe periodontal disease leads not only to tooth loss and decreased chewing function but also to decreased oral-related quality of life, such as pain during chewing, and increases the risk of new cases of oral frailty through decreased social and psychological functioning.

Moreover, in this study, chronic conditions (diabetes mellitus, osteoporosis, apoplexy) and polypharmacy were significantly more frequently observed among the participants with new-onset oral frailty. Polypharmacy is characterized by decreased medication adherence, inappropriate prescribing, and factors that increase the incidence of adverse drug events (e.g., old age, depression, living alone, and cognitive decline) that coexist with older adults with oral frailty [25,26]. Therefore, it cannot be denied that these problems may have led to new-onset oral frailty due to prolonged chronic disease. However, the results of the analysis excluding the effects of confounding factors showed no significant difference in the association between polypharmacy and new-onset of oral frailty and confirmed that the effect of severe periodontal disease was higher.

The results of this study showed that among six factors of oral frailty associated with severe periodontitis, the strongest correlation was found with decrease in the number of remaining teeth. This suggests a strong relationship between tooth loss caused by progression of periodontal disease and onset of oral frailty. Although no cross-sectional relationship was found at the baseline survey [4], the six-year follow-up demonstrated that periodontal disease increased the rate of new-onset oral frailty, which may have been influenced by this reduction in the number of remaining teeth and the various risks mentioned above. This also suggests that conventional measures for periodontal disease prevention or treatment are the first-step intervention to prevent worsening of multi-faceted oral functions such as chewing, swallowing, and oral motor skill. Therefore, it is considered important to detect and intervene in periodontal diseases at an early stage before oral functions deteriorate due to aging in order to promote health throughout the life course.

Given that periodontal disease is a chronic disease caused by microbial infections originating in periodontal plaque, daily oral hygiene and supplementary positive oral health behaviors such as having regular dental checkups and teeth cleaning are effective, basic measures for its prevention [27,28]. Moreover, severe periodontitis, whose impact on oral frailty was confirmed in this study, requires specialized treatment such as scaling and root planning, antimicrobial administration, and periodontal tissue regeneration in addition to those basic oral hygiene practices. In Japan, the “8020 Campaign”, a national health promotion campaign to keep 20 or more of one’s own teeth until the age of 80, has been implemented [29]. This campaign has led to widespread public awareness of the necessity of periodontal disease prevention, which has gradually increased the frequency of tooth brushing and the use of interdental cleaning tools [30]. However, demonstrating the link between periodontal disease and total oral function may increase public awareness of the importance of conventional oral health behaviors and risks of poor oral hygiene even more.

The findings of the present study highlight the importance of conventional approaches to periodontal disease prevention; however, additional measures must be taken to reinforce the importance of oral function and overall oral health maintenance hence-forth. Indeed, the efficacy of salivary gland massaging and various oral functional training such as tongue and swallowing exercises has been confirmed for treating decreased oral function [5,31]. However, it has also been reported that function decreases again after the end of the treatment and therapy intervention period, leading to recurrence [5]. There is a report that the effect of intervention can be maintained if patients can continue training at home after intervention [32], but it is challenging to get patients to make functional training a habit in their daily lives. Thus, in the approach to oral frailty prevention, it is important to emphasize existing conventional practices and habits of periodontal disease prevention behaviors as a foundation for maintaining overall oral health, including oral hygiene and function maintenance, and to also promote supplementary oral function exercises.

## 5. Strengths and Limitations

The evaluation of oral frailty in the present study involved assessments by dental professionals with objective indicators obtained through the utilization of specialized devices. The present study is the only field research to analyze new-onset oral frailty as an outcome and to consider the effects of changes over time, as the original oral frailty assessment was repeated four times with six years of follow-up. Furthermore, the study was conducted with a relatively large sample size and long follow-up period for a clinical study using dental indices. Additionally, the study was not conducted in typical dental checkup environments or dental clinics but in settings such as local community centers. These settings allowed for assessment of numerous confounding factors, objectively determined, in addition to the assessment of dental conditions. The greatest strength of this study thus lies in having evaluated a substantial number of community-dwelling older adults over time via numerous objective indicators rather than with questionnaires alone.

However, there are also several limitations to this study. First, we evaluated and corrected for possible confounding factors in the relationship between periodontal disease and oral frailty in this study. However, the possibility that there are unevaluated factors cannot be denied. Second, since the participants of this study were randomly selected in the baseline survey, the data are highly representative of the citizens. However, some participants were not followed-up, such as those who were absent from the follow-up survey, so survival bias may have affected the results.

## 6. Conclusions

This study revealed that severe periodontitis in community-dwelling older adults in-creased the risk of new-onset oral frailty. Thus, our findings suggest that periodontal disease prevention, which is conventionally implemented in the clinical setting, effectively not only improves the number of remaining teeth but also prevents oral frailty. To effectively prevent oral frailty, it is essential to prevent periodontal disease through adequate compliance to conventional oral care and dental checkups, and it is also important to provide additional oral function training.

## Figures and Tables

**Table 1:** Comparison of basic characteristics at baseline with and without new-onset of oral frailty.

	Total (*n* = 1234)	No-Onset (*n* = 949)	New-Onset (*n* = 285)	*P*
Basic attributes, Physical conditions				
Age, year	72.2 (±5.1)	71.7 (±4.8)	73.8 (±5.6)	<0.001
Sex, men	50.8%	51.2%	49.5%	0.607
BMI, kg/m^2^	23.0 (±3.0)	23.0 (±2.9)	22.9 (±3.3)	0.462
Education, year	12.9 (±2.8)	12.9 (±2.7)	12.7 (±2.8)	0.147
IADL	4.9 (±0.4)	4.9 (±0.4)	4.8 (±0.6)	0.086
Cognitive function, MMSE score	28.4 (±1.8)	28.4 (±1.7)	28.1 (±1.9)	0.021
GDS-15 score	2.4 (±2.7)	2.2 (±2.7)	2.7 (±2.7)	0.009
Yearly income (≤1.4 million yen)	55.6%	54.8%	58.2%	0.304
Polypharmacy (≥6 medications)	14.6%	12.9%	20.4%	0.002
Present chronic conditions				
Hypertension	41.4%	40.5%	44.6%	0.218
Diabetes mellitus	11.6%	10.5%	15.1%	0.035
Osteoporosis	9.3%	8.4%	12.6%	0.029
Dyslipidemia	39.9%	38.6%	44.2%	0.088
Malignant neoplasm	14.7%	14.2%	16.5%	0.344
Heart disease	15.5%	14.9%	17.5%	0.272
Apoplexy	5.7%	4.7%	8.8%	0.010
Oral frailty component				
Number of remaining teeth	23.0 (±6.5)	24.4 (±5.2)	18.2 (±7.7)	<0.001
Chewing ability	18.4 (±5.9)	19.0 (±5.9)	16.5 (±5.6)	<0.001
Tongue pressure	31.4 (±7.3)	31.8 (±7.2)	29.9 (±7.4)	<0.001
Articulatory oral motor skill (“ta” times/s)	6.2 (±0.8)	6.3 (±0.8)	6.0 (±0.9)	0.001
Difficulties eating tough foods (yes)	8.7%	6.7%	15.1%	<0.001
Difficulties in swallowing on tea or soup (yes)	14.3%	11.8%	22.5%	<0.001
Other Oral status				
Number of functioning teeth	27.3 (±2.3)	27.4 (±2.2)	26.8 (±2.5)	<0.001
Oral wettability (<3 mm classified as dry mouth)	34.2%	34.3%	33.9%	0.899

Notes: BMI, body mass index; IADL, instrumental activities of daily living; MMSE, mini-mental state examination; GDS-15, geriatric depression scale—15.

**Table 2:** Comparison of periodontal status at baseline with and without new-onset of oral frailty.

	Total (*n* = 1234)	No-Onset (*n* = 949)	New-Onset (*n* = 285)	*P*
Community Periodontal Index (CPI)				
Score 0	17 (1.4%)	12 (1.3%)	5 (1.8%)	0.187
Score 1	17 (1.4%)	12 (1.3%)	5 (1.8%)	
Score 2	281 (22.8%)	217 (22.9%)	64 (22.5%)	
Score 3 (moderate periodontitis)	439 (35.5%)	353 (37.2%)	86 (30.2%)	
Score 4 (severe periodontitis)	480 (38.9%)	355 (37.4%)	125 (43.9%)	

**Table 3:** Relationship between periodontal status at baseline and new-onset of oral frailty.

	*n* (%) ^a^	Unadjusted Hazard Ratio (95% CI)		*p*	Adjusted Hazard Ratio (95% CI) ^b^		*P*
The number of participants	285/1234 (23.1%)						
Community Periodontal Index (CPI)							
≤2	74/315 (23.5%)	1.00			1.00		
≥3 (moderate periodontitis)	211/919 (23.0%)	1.02	(0.78–1.33)	0.892	1.09	(0.83–1.44)	0.530
≤3	160/754 (21.2%)	1.00			1.00		
4 (severe periodontitis)	125/480 (26.0%)	1.38	(1.09–1.74)	0.007	1.42	(1.12–1.81)	0.005

a, *n* (%) indicates “the number of individuals with new-onset oral frailty/the number of total participants”; b, the hazard ratios were adjusted by the potential confounders as follows: age, sex, body mass index, number of teeth, education, instrumental activates daily living, cognition, depressive symptoms, low yearly income, present chronic conditions, polypharmacy, and oral wettability.

## Data Availability

Not applicable.
